# Dark Septate Endophytic Fungi Help Tomato to Acquire Nutrients from Ground Plant Material

**DOI:** 10.3389/fmicb.2017.02437

**Published:** 2017-12-11

**Authors:** Carlos Vergara, Karla E. C. Araujo, Segundo Urquiaga, Nivaldo Schultz, Fabiano de Carvalho Balieiro, Peter S. Medeiros, Leandro A. Santos, Gustavo R. Xavier, Jerri E. Zilli

**Affiliations:** ^1^Departamento de Ciências do Solo, Universidade Federal Rural do Rio de Janeiro, Seropédica, Brazil; ^2^Departamento de Fitotecnia, Universidade Federal Rural do Rio de Janeiro, Seropédica, Brazil; ^3^Embrapa Agrobiologia, Seropédica, Brazil; ^4^Embrapa Solos, Seropédica, Brazil

**Keywords:** *Solanum lycopersicum* (L.), ^15^N, DSE fungi, nutrient recovery efficiency, potassium, phosphorus, *Canavalia ensiformis* (L.) -^15^N

## Abstract

Dark septate endophytic (DSE) fungi are facultative biotrophs that associate with hundreds of plant species, contributing to their growth. These fungi may therefore aid in the search for sustainable agricultural practices. However, several ecological functions of DSE fungi need further clarification. The present study investigated the effects of DSE fungi inoculation on nutrient recovery efficiency, nutrient accumulation, and growth of tomato plants fertilized with organic and inorganic N sources. Two experiments were carried out under greenhouse conditions in a randomized blocks design, with five replicates of tomato seedlings grown in pots filled with non-sterile sandy soil. Tomato seedlings (cv. Santa Clara I-5300) inoculated with DSE fungi (isolates A101, A104, and A105) and without DSE fungi (control) were transplanted to pots filled with 12 kg of soil which had previously received finely ground plant material [*Canavalia ensiformis* (L.)] that was shoot enriched with 0.7 atom % ^15^N (organic N source experiment) or ammonium sulfate-^15^N enriched with 1 atom % ^15^N (mineral N source experiment). Growth indicators, nutrient content, amount of nitrogen (N) in the plant derived from ammonium sulfate-^15^N or *C. ensiformis-*^15^N, and recovery efficiency of ^15^N, P, and K by plants were quantified 50 days after transplanting. The treatment inoculated with DSE fungi and supplied with an organic N source showed significantly higher recovery efficiency of ^15^N, P, and K. In addition, the ^15^N, N, P, K, Ca, Mg, Fe, Mn, and Zn content, plant height, leaf number, leaf area (only for the A104 inoculation), and shoot dry matter increased. In contrast, the only positive effects observed in the presence of an inorganic N source were fertilizer-K recovery efficiency, content of K, and leaf area when inoculated with the fungus A104. Inoculation with A101, A104, and A105 promoted the growth of tomato using organic N source (finely ground *C. ensiformis-*^15^N plant material).

## Introduction

In agriculture, especially in low-input or organic cultivation, plants can benefit from interactions with microorganisms, such as arbuscular mycorrhizal fungi (AMF) (He and Nara, 2007). In this case, their extraradicular mycelium provides a wide nutrient interception area and a high nutrient uptake efficiency, decreasing demands on applied fertilizers and reducing soil nutrient loss, in addition to improving plant nutrition and growth (Hogberg, 1989; Finlay and Söderström, 1992; He and Nara, 2007; Cavagnaro et al., 2015). Furthermore, evidence exists that AMF can acquire nutrients from organic matter, probably after its mineralization, and transfer them to the host plant (Hodge et al., 2001; Hodge and Fitter, 2010). However, fundamental studies and production of a high quality inoculum (free of impurities) of AMF have been limited by the fact that such fungi are obligate biotrophs, requiring establishment of effective associations with metabolically active roots for their development and multiplication (Sylvia and Jarstfer, 1994).

Dark septate endophytic (DSE) fungi are quite diverse facultative biotrophic ascomycetes characterized by dark pigmentation, melanized septate hyphae, and formation of microsclerotia inside the plant roots. Such fungi are capable of colonizing root tissues intra and intercellularly of more than 600 plant species [including *Solanum lycopersicum* (L.) and those non-mycorrhizal ones] without causing pathologies. They can also act as plant growth promoters (Jumpponen and Trappe, 1998; Upson et al., 2009; Andrade-Linares et al., 2011a,b; Yuan et al., 2011; Mahmoud and Narisawa, 2013).

Tomato plants belongs to the family *Solanaceae* and have been determined to be the second most economically important vegetable worldwide after the potato (Foolad, 2004; Reis Filgueira, 2008). Tomato fruits are an important source of lycopene, which exhibits antioxidant, hypolipidemic, and anti-carcinogenic properties (Viuda-Martos et al., 2014). In addition, tomatoes are cholesterol free and rich in mineral salts, vitamins A and C, and fibers (Block et al., 1992; Gerster, 1997; Rao and Agarwal, 2000; Viuda-Martos et al., 2014).

Studies have suggested that DSE fungi are capable of accessing complex carbon (C), nitrogen (N), and phosphorus (P) compounds within the soil when associated with plants (Mandyam and Jumpponen, 2005), making nutrients available to their host plant (Mandyam, 2008). This is probably because they produce hydrolytic enzymes, which induce release of nutrients absorbed by plants. Additionally, organic compounds taken up by DSE fungi, including amino acids and small peptides, are transferred directly to host plants (Jumpponen and Trappe, 1998), resulting in better use of organic nutrient sources (Reeve et al., 2008).

In previous studies, three isolates of DSE fungi, A101, A104, and A105 obtained from wild rice (*Oryza glumaepatula* [Steud.]) were determined by ITS phylogeny to belong to the order *Pleosporales* (suborder *Massarineae*) (A104 and A105) and to an unknown taxon (A101) (Ribeiro, 2011; Vergara et al., 2017). These fungi colonize wild [*Oryza glumaepatula* (Steud.)] and commercial [*Oryza sativa* (L.)] rice and tomato (cv. Santa Clara I-5300) plants, with no apparent symptoms of disease (Ribeiro, 2011; Vergara et al., 2017). Furthermore, a neutral effect was observed in the N content of tomato plants inoculated with these fungi when NH_4_NO_3_ (an inorganic source) was supplied to the plants.

In the present study, the effects of inoculation with three DSE fungi on tomato plants (cv. Santa Clara I-5300) was evaluated in non-sterile sand soil in greenhouse conditions. Plant growth, nutrient accumulation (N, P, K, Ca, Mg, Fe, Mn, and Zn), the amount of N in the plants derived from ammonium sulfate-^15^N or from a finely ground legume *Canavalia ensiformis*-^15^N, and recovery efficiency of ^15^N, P, and K by plants were determined. Thus, the present study investigated the effects of DSE inoculation on nutrient recovery efficiency, which is the proportion of a given fertilizer nutrient taken up by a plant, nutrient accumulation, and growth of tomato plants fertilized with organic and inorganic N sources.

## Materials and methods

*Canavalia ensiformi*s (L.), a plant that is extensively used in tropical agriculture as a green manure for nutritional enrichment of soils (Rodrigues et al., 2004), and ammonium sulfate were used as N source for tomato plants inoculated with DSE fungi. To study the differences between these N sources, two experiments were conducted in parallel: (i) an experiment with the inorganic N source, ammonium sulfate; and (ii) an experiment with the organic N source, *C. ensiformis*. Ammonium sulfate is an important source of N, but also provides sulfur for plants. On the other hand, green manure *C. ensiformis* (a legume) contributes considerable amounts of N to the soil-plant system due to its association with N_2_ fixing bacteria (Perin et al., 2003). *C. ensiformis* also maintains soil moisture (Tejada et al., 2008) and mobilizes nutrients such as P and some micronutrients (Lambers et al., 2015).

### Fertilization and liming of the soil for both experiments

For both experiment, soil samples were collected from an organic production system located at Seropédica Municipality, RJ, Brazil, at 0–20 cm depth. The soil was classified as Haplic Planosol (according to Brazilian Soil Taxonomy, or Planosol, under World Reference Base-FAO). Soil chemical analysis showed the following properties: pH = 5.61 in water; exchangeable Al^3+^ = 0.0 and H+Al = 2.95 cmol_c_ dm^−3^ (centimoles of charge per dm^3^ soil); Ca^+2^ = 1.21 and Mg^+2^ = 0.65 cmol_c_ dm^−3^; available *P* = 10.27 and K^+^ = 63.92 mg L^−1^; total *N* = 0.07% and *C* = 0.54%. The soil was classified as sandy soil (3% clay, 5% silt, and 92% sandy). For each experimental unit, a pot with a 14 L capacity was filled with 12 kg of a sieved and homogenized soil sample. Two months after lime addition (equivalent of 1.94 t ha^−1^; *MineralCal*) to correct for Ca^+2^ and Mg^+2^ deficiencies, the soil was fertilized with the equivalent of 320 kg P_2_O_5_ ha^−1^ (simple superphosphate), 68 kg K_2_O ha^−1^ (potassium sulfate), and 30 kg ha^−1^ micronutrient fertilizer as F.T.E BR-12 (fritted trace elements), according to recommendations for tomato crops (Macebo et al., 2013). Potassium was applied in two stages, 25 (27 kg K_2_O ha^−1^) and 40 (41 kg K_2_O ha^−1^) days after transplanting (DAT).

### Nitrogen fertilization labeled by ^15^N

Ammonium sulfate was used as an inorganic fertilizer source. Application of ammonium sulfate enriched with 1 atom % ^15^N (ammonium sulfate-^15^N) was split into two equal levels (0.428 g per pot, equivalent to 15 kg ha^−1^ N), at 25 and 45 DAT (Macebo et al., 2013). On the day of fertilizer-^15^N application, ^15^N-labeled ammonium sulfate was dissolved in distilled water and 500 mL of the homogenized solution was evenly distributed in pots for soil labeling standardization by soil drenching.

For the experiment with an organic N source, dry, finely ground shoot biomass of *Canavalia ensiformis* (L.) was used. *C. ensiformis* plants were previously grown in ^15^N-enriched soil for use as green manure, and their dry shoot (dried 72 h at 65°C) enriched with 0.7 atom % ^15^N (*C. ensiformis*-^15^N) was sampled at around 60–70 days after germination (flowering period). The dry shoot of *C. ensiformis* was finely ground and then sterilized by gamma irradiation (25 kGy). Macro- (g kg^−1^) and micronutrient (mg kg^−1^) concentrations for *C. ensiformis*-^15^N were: N = 23.8; P = 2.0; K = 5.8; Ca = 12.3; Mg = 3.2; S = 1.9; Cu = 10.0; Fe = 792.0; Zn = 39.0; Mn = 50.0; B = 27.0; and C = 38.2%. Each pot was filled with 12 kg soil receiving 37.81 g of finely dry ground biomass of *C. ensiformis*, equivalent to 150 kg N ha^−1^, which was applied and homogenized before planting.

### Inoculum preparation and inoculation

The fungal isolates used in the two experiments were obtained from *O. glumaepatula* and identified through ITS phylogeny (Ribeiro, 2011; Vergara et al., 2017). These fungi are deposited in the Centro de Recursos Biológicos Johanna Döbereiner (www.embrapa.br/agrobiologia/crb-jd) culture collection (A101, A104, and A105). The ITS region sequences are deposited in GenBank (KR817246 = A101, KR817249 = A104, and KR817250 = A105). The inoculum was obtained according to Andrade-Linares et al. (2011b), with some modifications. Each isolate was grown in a 300 mL Erlenmeyer flask containing 150 mL potato dextrose agar (PDA) medium for 2 weeks at 28°C under 80 rpm shaking. The fresh mycelium was filtered and washed with sterile distilled water until the liquid became clear to avoid transfer of any material from the PDA medium to the inoculum. Then, the mycelium was weighed and part of it was mixed with sterile distilled water for 1 min at minimum speed using a mixer (Arno Optimix Plus, model LN27, Brazil) operating at laminar flow to prevent contamination. The viability of each isolate was verified by plating the mycelium in the PDA medium. For inoculation, suspensions were adjusted with sterile distilled water to a concentration of 1% (*w/v*).

### Experimental design and growth conditions

Two experiments with tomato seedlings were carried out in parallel, in randomized blocks, with five replicates with one plant each (*n* = 5), under greenhouse conditions at Embrapa Agrobiologia, in Seropédica Municipality, RJ, Brazil. For both experiments, treatments consisted of tomato (*S. lycopersicum* cv. Santa Clara I-5300) plants grown with no inoculation (control) and inoculated with DSE fungi (A101, A104, and A105). All treatments using an inorganic N source received ammonium sulfate-^15^N as the sole N source, while treatments using organic N source received *C. ensiformis*-^15^N. Santa Clara I-5300 is an indeterminate tomato cultivar belonging to the Santa Cruz group, which has been grown in the Brazilian Center-South region since 1940 (Reis Filgueira, 2008). Tomato seeds were washed with 70% alcohol for 3 min and disinfected with 2.5% sodium hypochlorite for 3 min, followed by eight successive washes in sterile distilled water. Then, seeds were pre-germinated in water agar (8 g L^−1^) at 28°C to select homogenous plants for both experiments.

Tomato seedlings showing 1–2 leaves were inoculated with DSE fungi by root immersion in the mycelial suspension (1% *w*/*v*), while control plants only received sterile distilled water. The soil (12 kg) of inoculation treatments was also drenched by the 500 mL suspension (1% *w*/*v*) containing the fresh mycelium, while the control group only received sterile distilled water. Pots were watered daily with 500 mL distilled water to maintain soil moisture near field capacity.

### Colonization and pathogenicity observations

To confirm whether the three DSE fungi colonized the inner roots endophytically, the roots of tomato plants inoculated with A101, A104, and A105 were cleaned and fixed in 50% ethanol. Then the samples were soaked in 2.5% potassium hydroxide (KOH) overnight. Subsequently, roots were acidified with 1% hydrochloric acid overnight at room temperature, followed by staining with 0.01% (w/v) methyl blue (a mixture of 10:9:1 glycerol/distilled water/hydrochloric acid; Phillips and Hayman, 1970). Finally, roots were placed into 50% ethanol to de-stain. Root sections (approximately 3 cm) were placed on slides with glycerin and hyphal structures were viewed with an Axioplan light microscope (Carl Zeiss, Jena, 151 Germany) equipped with an Axiocam MRC5 digital camera (Carl Zeiss). To prepare cross-sections of colonized roots for light microscopy, samples were dehydrated twice in an ethanol series of 70, 90, and 100% for 1 h each. After dehydration, the colonized roots were infiltrated with historesin (Leica, Wetzlar, Germany) and 100% ethanol (1:1, v/v) for 12 h and then with 100% historesin for 24 h before being embedded in historesin. Sections (approximately 5 μm) were obtained using a rotary microtome (Leica) (Vergara et al., 2017). Samples were observed and images were analyzed as described above. Symptoms were evaluated on scale of 0–3 (0: no visible symptoms; 1: light yellowing; 2: yellowing and late growth; 3: wilting or death) (Diene et al., 2013; Mahmoud and Narisawa, 2013).

### Measurements

Aboveground dry biomass, plant height, stem diameter, leaf number, and total leaf area (LI-3100C area meter, LI-COR, Nebraska, USA) of tomato plants were measured at 50 DAT in both experiments. Dry shoot tissue (dried at 65°C, 72 h) was ground in a Wiley-type laboratory mill (<40 mesh) followed by a rolling mill to decrease sample grain size (Smith and Um, 1990).

The concentrations of N, P, K, Ca, Mg, Zn, Fe, Mn, and the ^15^N abundance (atom % ^15^N excess) were determined in the aboveground tissues. Micronutrient concentrations were quantified in an aqua regia extract (ISO 12914, 2012) by a plasma detector (PerkinElmer® Optima™ 8,300), while macronutrient concentrations were obtained according to Tedesco (1982).

^15^N abundance was quantified using continuous-flow isotope ratio mass spectrometry (Finnigan DeltaPlus mass spectrometer coupled to the output of a Carlo Erba EA 1108 total C and N analyzer—Finnigan MAT, Bremen, Germany) (Boddey et al., 1994). Macro- and micronutrients content (mg plant^−1^) were estimated according to equation (1), using the concentration and dry matter (mg plant^−1^) accumulated by the tomato plants in each treatment.

(1) $$\begin{array}{l} \text{Nutrient content}\\ \left(\text{mg }{\text{plant}}^{-\text{1}}\right)\;=100÷[\text{Nutrient concentration }\left(\%\right)\\ \;\times \text{ Dry matter}(\text{mg }{\text{plant}}^{-\text{1}})] \end{array}$$

The fraction (%) and amount (mg plant^−1^) of N in the plant derived from fertilizer-^15^N or finely ground *C. ensiformis*-^15^N, as well as the recovery efficiency of ^15^N, were measured using Equations (2–4), according to International Atomic Energy Agency (2001). The atoms % ^15^N excess was obtained by the difference between ^15^N abundance in plants and the ^15^N natural abundance in the air (0.3663% atoms).

(2) Fraction of N in the plant derived from N15−labelled fertilizer (%)​=​100 × ​(atom %N15 excessplant atom% N15 excessfertilizer )

(3) Amount of N in the plant derived from N15−labelled fertilizer (mg plant−1)=100÷[Fraction of N in the plant derived from N15                                                                                                                                     −labelled fertilizer (%)×Nitrogen content (mg plant−1)]

(4) N recovery efficiency=Amount of N in the plant derived from N15−labelled fertilizer (mg plant−1)Amount of applied N as N15−labelled fertilizer (mg pot−1)×100

The apparent nutrient recovery efficiency of P and K in shoots was calculated according to Baligar and Fageria (1997). Apparent nutrient recovery efficiency reflects the efficiency of plants obtaining nutrients from soil per unit of nutrient applied, as described by Equation (5).

(5) $$\begin{array}{l} \text{Apparent nutrient recovery efficiency }\left(\%\right)=100\times [(\text{nutrient absorbed by plants from fertilized plots},\text{ mg }{\text{plant}}^{-\text{1}}\\ \;-\text{ nutrient absorbed by plants from unfertilized plots},\text{ mg }{\text{plant}}^{-\text{1}})\\ \;÷\text{ amount of nutrient applied},\text{ mg }{\text{plant}}^{-\text{1}}] \end{array}$$

### Statistical analysis

For each experiment, data were individually submitted to tests for homogeneity (Bartlett) and normality (Shapiro-Wilk) of variances. Because N recovery efficiency from the mineral N source did not meet the assumptions of variance analysis, box-cox transformation was applied (Box and Cox, 1964; Venables and Ripley, 2002; Osborne, 2010). Then the data variance was measured (ANOVA). When ANOVA indicated significant differences, means of treatments were separated through the least significant difference (LSD) calculated by the *t*-test (*p* < 0.05). The software R-project version R 3.4.1 (R Development Core Team, 2017) with RStudio (version 1.0.153 and the package agricolae; de Mendiburu, 2017) was used for analysis and data are shown as mean ± standard error.

## Results

### Colonization and pathogenicity observations

**Figure 1:**
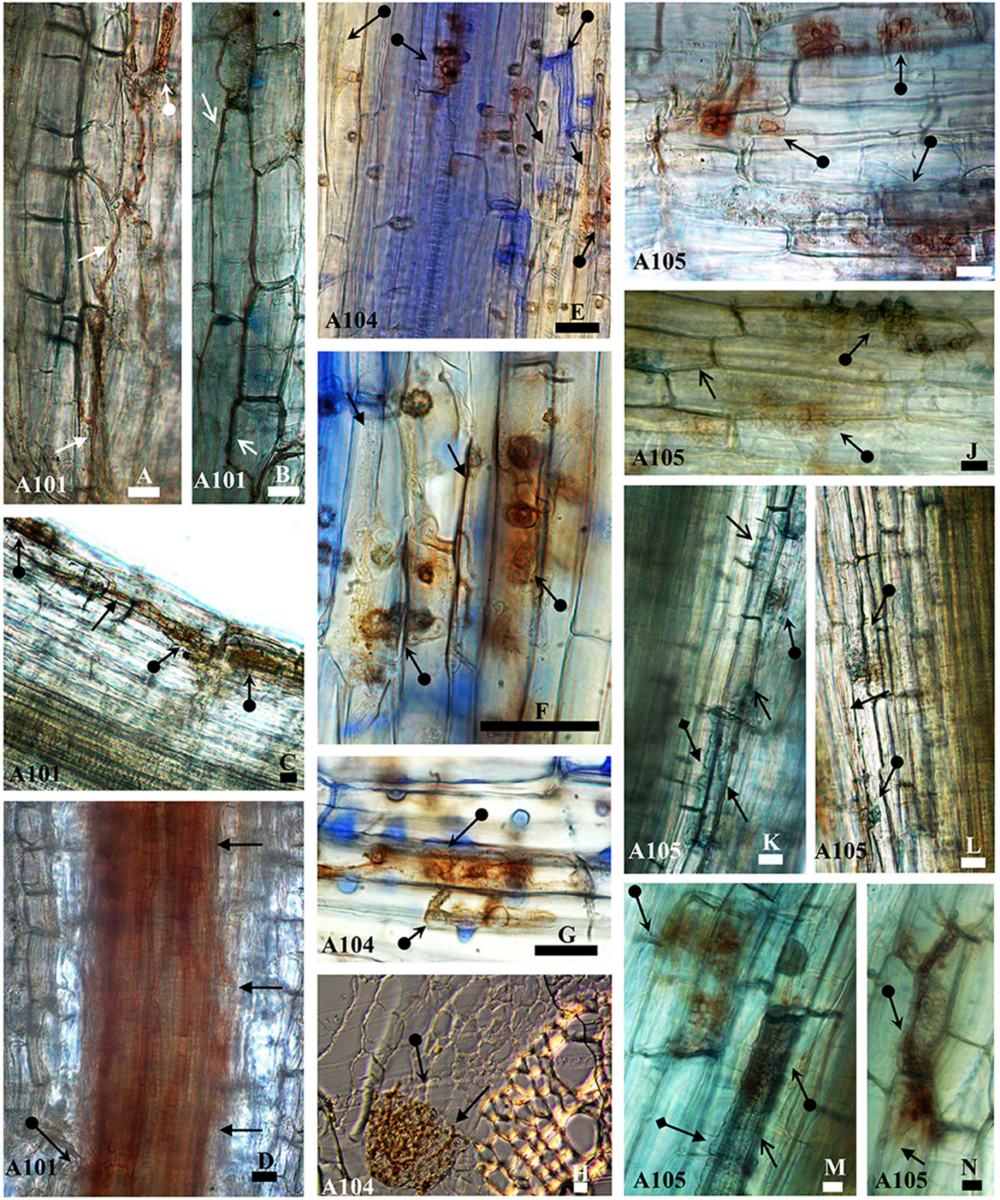
Morphological aspects of tomato roots (Santa Clara I-5300 variety) inoculated with the fungi A101, A104, and A105 at 50 DAT. Melanized septate hyphae surround (arrowheads) or not (arrow) cells in the epidermis **(B, J, M**, and **A, E, F, N**, respectively) and in the cortex (**K, C, and, H, L**, respectively) and in the region between the cortex and vascular bundle **(D)**. Microsclerotia-like structures (arrow with circle at the base) formed by the fungi in the epidermis **(A, E–G,I,J,M**, and **N)**, in the cortex **(C, H, K, L)**, and in the region between cortex and vascular bundle **(D)**. Hyaline vesicles (arrow with diamond base) formed in the epidermis **(M)** and the cortex **(K)**. Samples were stained with 0.01% methyl blue, except **(H)**, which was obtained from an unstained cross section. Bar = 20 μm.

The three isolates (A101, A104, and A105) (Figures 1A–N) colonized the root tissue of tomato plants with hyphae colonizing epidermis (Figures 1A,B,E,F,J,M,N), cortex (Figures 1C,H,K,L), and forming microsclerotia-like structures (Figures 1A,C,E–N), with no symptoms of apparent disease. In addition, the melanized septate hyphae of A101 (Figure 1B) and A105 (Figures 1J,M) fungi surrounded cells in the epidermis. A105 was also able to surround cells in the cortex (Figure 1K). The three fungi differed in their colonization patterns. While A101 colonized the region between the cortex and vascular bundle (Figure 1D), A104 formed a network in the epidermis connecting several cells (Figure 1E with details in Figure 1F), and A105 formed abundant vesicles in the epidermis (Figure 1M) and cortex (Figure 1K).

### DSE inoculation effects on plant growth

The three DSE isolates (A101, A104, and A105) promoted tomato growth without causing any typical disease symptoms using finely ground *C. ensiformis*-^15^N as the organic N source. Tomato plants exhibited significant increases in aboveground dry biomass (25, 34, and 41% increases for A101, A104, and A105, respectively), plant height, and leaf number, relative to the uninoculated treatment (Table 1). A104 stood out from other isolates, because the leaf number and total leaf area of inoculated plants also exhibited significant increases of 50 and 67%, respectively, in comparison to uninoculated control plants (Table 1). However, inoculation had no effect on stem diameter. Likewise, DSE-tomato interaction had no effect on aboveground dry biomass, leaf number, and stem diameter of the tomato plants when inorganic N was supplied as ammonium sulfate-^15^N (Table 2). However, significant differences were detected for total leaf area, especially in the DSE-tomato interaction compared with control plants (Table 2).

**Table 1:** Growth indicators of tomato plants at 50 DAT with no plant inoculation (control) and inoculation with dark septate endophyte (DSE) fungi and fertilized with an organic N source (finely ground *Canavalia ensiformis* [L.]-^15^N).

**Treatment**	**Aboveground dry biomass (g plant^−1^)**	**Plant height (cm plant^−1^)**	**Leaf number (unit plant^−1^)**	**Total leaf area (cm^2^ plant^−1^)**	**Stem diameter (cm plant^−1^)**
Control	10.8 ± 0.05b	53 ± 0.41b	14 ± 0.20c	1, 885 ± 12b	10 ± 0.12
A101	14.5 ± 0.34a	63 ± 3.35a	18 ± 0.47b	2, 182 ± 129b	11 ± 0.16
A104	13.6 ± 0.90a	63 ± 2.95*a*	21 ± 1.52a	3, 155 ± 248a	11 ± 0.33
A105	15.2 ± 1.03a	66 ± 2.25a	18 ± 1.25b	2, 095 ± 41b	11 ± 0.68
CV (%)	10.37	8.23	11.46	12.15	7.24

*Means ± SE (n = 5) followed by the same lowercase letter in the column indicates results do not differ by the LSD t-test (p < 0.05). The absence of letters indicates no significant difference by the F-test (p < 0.05). DAT, days after transplanting; SE, standard error*.

**Table 2:** Growth indicators of tomato plants at 50 DAT with no plant inoculation (control) and inoculation with DSE fungi and fertilization with an inorganic N source (ammonium sulfate-^15^N).

**Treatment**	**Aboveground dry biomass (g plant^−1^)**	**Plant height (cm plant^−1^)**	**Leaf number (unit plant^−1^)**	**Total leaf area (cm^2^ plant^−1^)**	**Stem diameter (mm plant^−1^)**
Control	16.9 ± 0.6	63 ± 1.82	14 ± 0.66	1, 727 ± 61b	10 ± 0.60
A101	17.1 ± 0.6	68 ± 1.38	15 ± 0.73	2, 041 ± 59a	10 ± 0.28
A104	18.4 ± 0.5	67 ± 2.07	16 ± 0.51	2, 044 ± 132a	10 ± 0.26
A105	17.4 ± 0.5	67 ± 0.81	16 ± 0.40	2, 016 ± 61a	11 ± 0.56
CV (%)	12.13	5.37	8.6	9.61	10.21

*Means ± SE (n = 5) followed by the same lowercase letter in the column indicates results do not differ by the LSD t-test (p < 0.05). The absence of letters indicates no significant difference by the F-test (p < 0.05). DAT, days after germination. SE, standard error*.

### Fertilizer-^15^N, -P, and -K recovery efficiency

**Figure 2:**
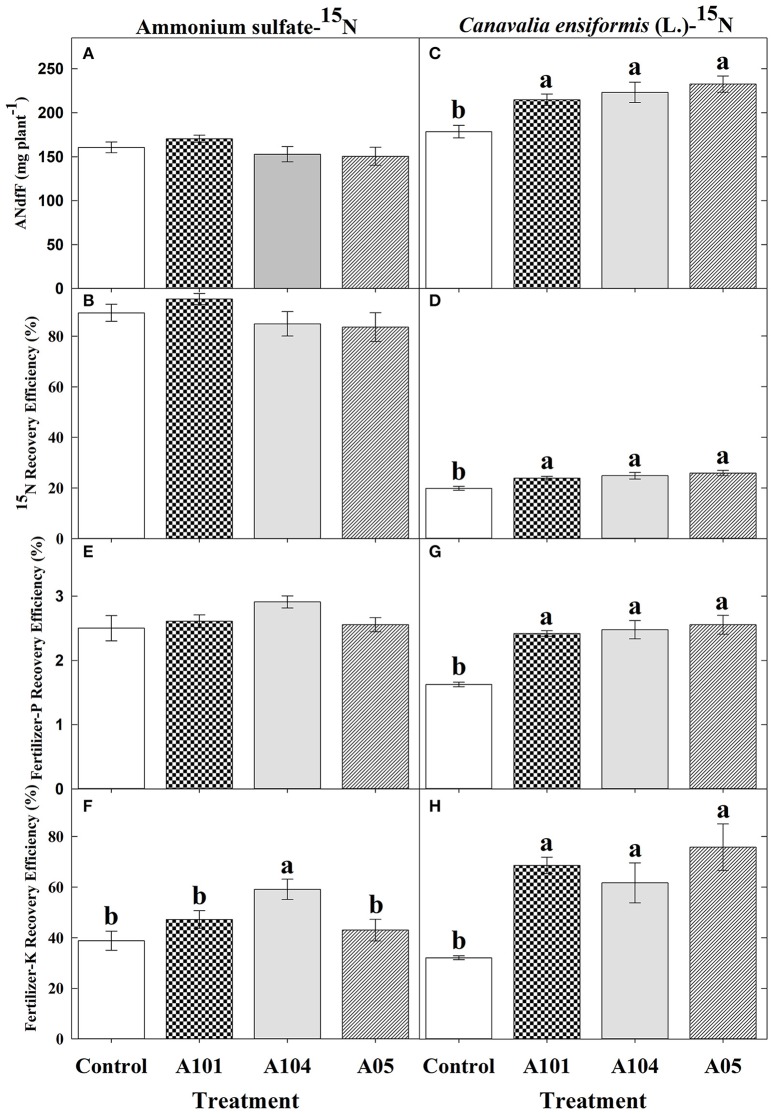
Amount of nitrogen (N) in the plant derived from ammonium sulfate-^15^N **(A)** or finely ground *C. ensiformis* (L.)-^15^N **(C)**, recovery efficiency of ammonium sulfate-^15^N **(B)** or finely ground *C. ensiformis*-^15^N **(D)**, apparent recovery efficiency of fertilizer-P [**(E)** for ammonium sulfate-^15^N and **(G)** for *C. ensifomis*-^15^N] and -K [**(F)** for ammonium sulfate-^15^N and **(H)** for *C. ensifomis*-^15^N] at 50 DAT by tomato (cv. Santa Clara I-5300) plants with no inoculation (control) and inoculated with dark septate endophytic (DSE) fungi A101, A104, and A105, and fertilized with an inorganic (ammonium sulfate-^15^N) or an organic (finely ground *C. ensiformis* [L.]-^15^N) N source. Among treatments, values followed by the same lowercase letter do not differ by LSD *t*-test (*p* < 0.05). The absence of letters indicates no significant difference by the *F*-test (*p* < 0.05). Error bars represent standard error of mean (*n* = 5).

In general, both uninoculated and inoculated tomato plants recovered N from the inorganic source (ammonium sulfate-^15^N) more efficiently than from the organic source (finely ground *C. ensiformis*-^15^N) (Figures 2A–D). However, the effect of DSE inoculation on ^15^N recovered was evident (and significant) only when tomato plants were fertilized by finely ground *C. ensiformis*-^15^N (Figures 2A–D). The amount of nitrogen in the plant derived from *C. ensiformis*-^15^N and the recovery efficiency of ^15^N from this source significantly increased in the DSE-tomato plants (by 20–30%) compared to the uninoculated control plants (Figures 2C,D). Conversely, tomato plants fertilized with the inorganic source showed no significant differences among the treatments for the amount of nitrogen in the plant derived from fertilizer-^15^N and for the fertilizer-^15^N recovery efficiency.

In the presence of *C. ensiformis*-^15^N, DSE isolates A101, A104, and A105 significantly increased the fertilizer-P and -K apparent recovery efficiency by 49, 52, and 57% and by 114, 92, and 136%, respectively, relative to uninoculated control plants (Figure 2E–H). However, in the presence of ammonium sulfate-^15^N, only the fertilizer-K apparent recovery efficiency was increased by 52% with the fungus A104.

### Macro- and micronutrients accumulation

**Figure 3:**
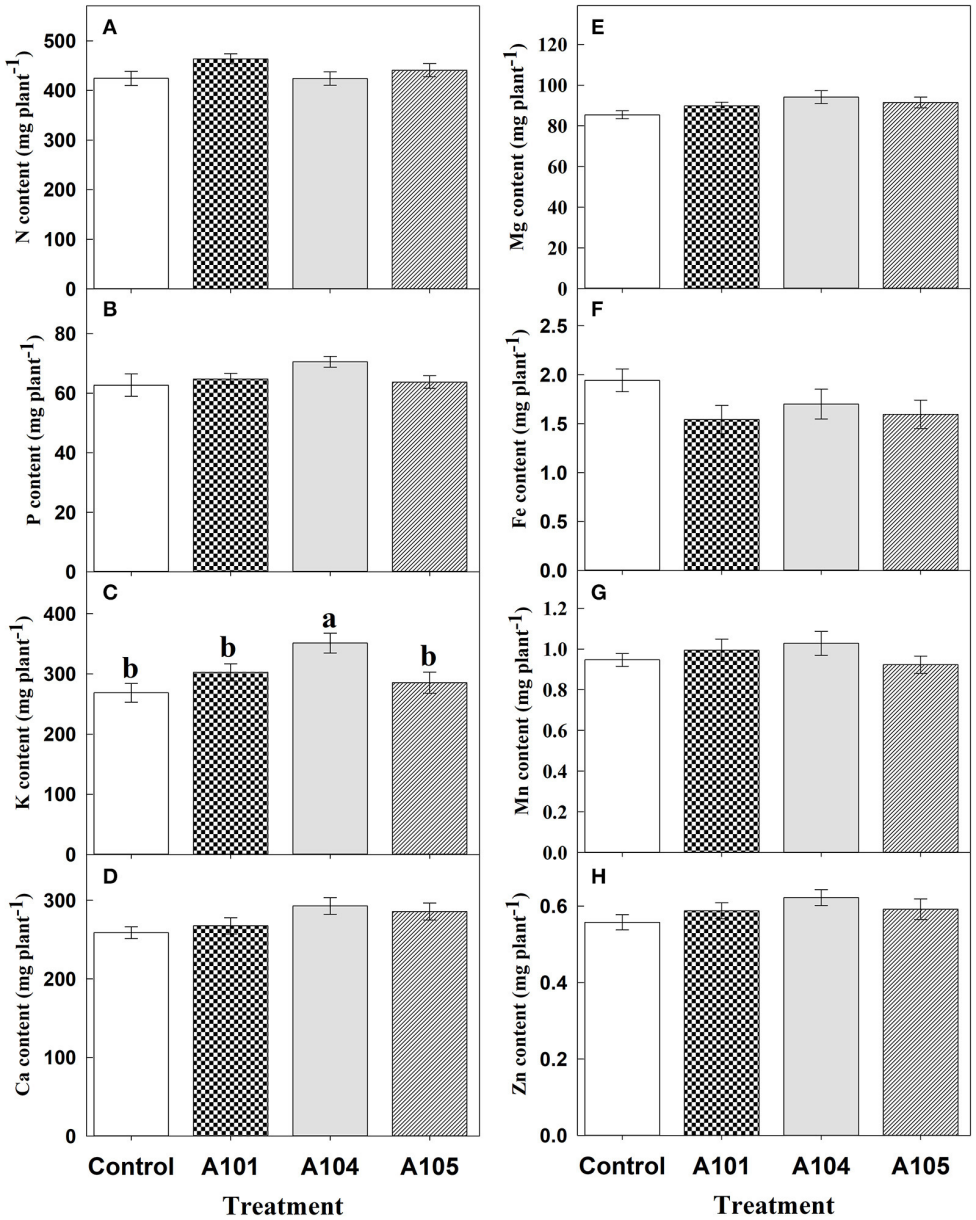
Contents of N **(A)**, P **(B)**, K **(C)**, Ca **(D)**, Mg **(E)**, Fe **(F)**, Mn **(G)**, and Zn **(H)** at 50 DAT for tomato (cv. Santa Clara I-5300) plants with no inoculation (control) and inoculated with dark septate endophytic (DSE) fungi A101, A104, and A105, and fertilized with inorganic N source (ammonium sulfate-^15^N). Among the treatments, values followed by the same lowercase letter do not differ by LSD *t*-test (*p* < 0.05). The absence of letters indicates no significant difference by the *F*-test (*p* < 0.05). Error bars represent standard error of mean (*n* = 5).

**Figure 4:**
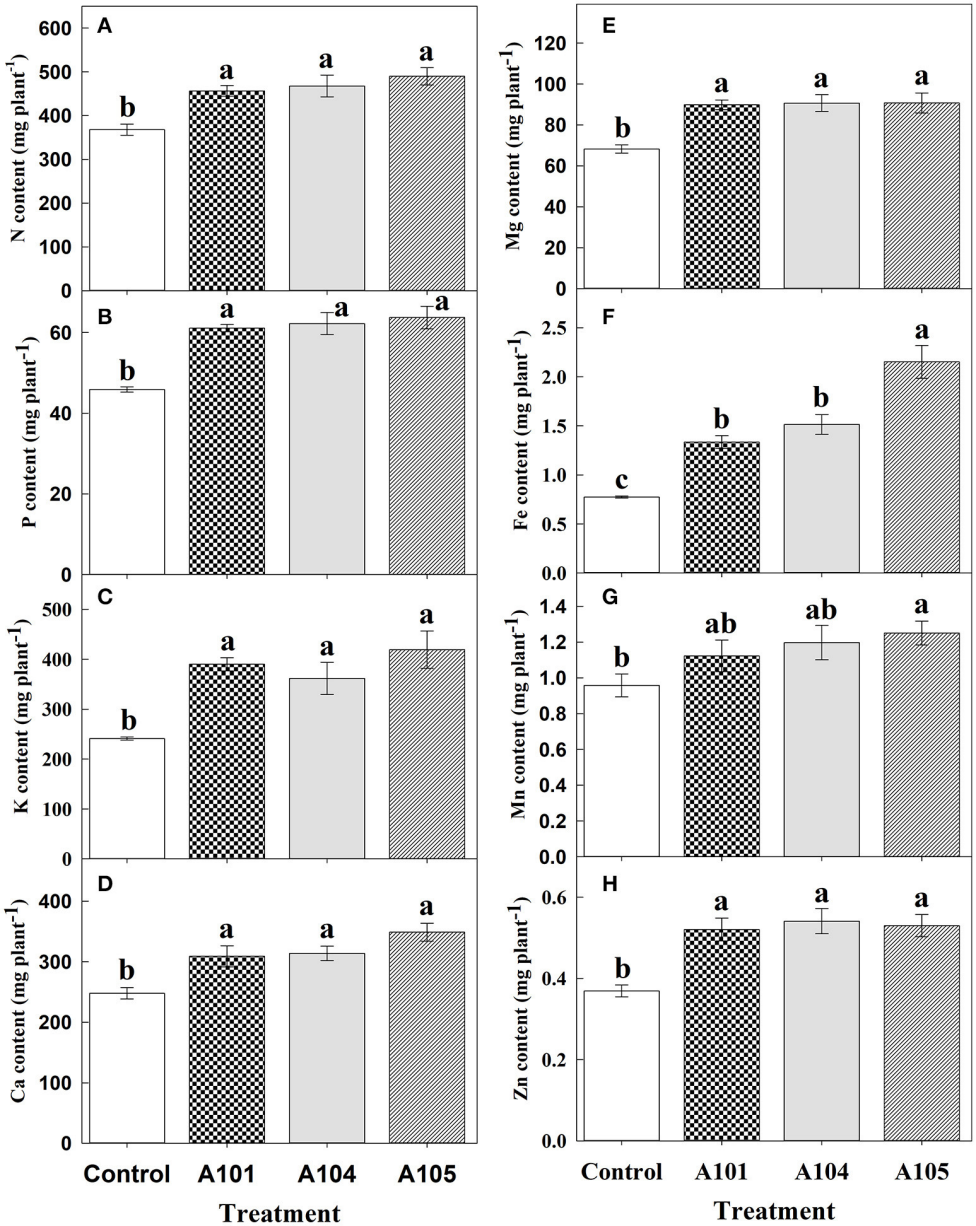
Contents of N **(A)**, P **(B)**, K **(C)**, Ca **(D)**, Mg **(E)**, Fe **(F)**, Mn **(G)**, and Zn **(H)** at 50 DAT for tomato (cv. Santa Clara I-5300) plants with no inoculation (control) and inoculated with dark septate endophytic (DSE) fungi A101, A104, and A105, and fertilized with organic N source (finely ground *Canavalia ensiformis* [L.]-^15^N). Among the treatments, values followed by the same lowercase letter do not differ by LSD *t*-test (*p* < 0.05). Error bars represent standard error of mean (*n* = 5).

Inoculation treatments did not affect the contents of N, P, Ca, Mg, Fe, Mn, and Zn (Figures 3A,B,D–H) when ammonium sulfate-^15^N was applied, which corroborates the observed fertilizer-^15^N (Figure 2B) and -P (Figure 2E) recovery efficiencies. However, inoculation with the fungus A104 led to a significantly higher K accumulation (an increase of about 30%) compared to the control and other treatments (Figure 3C), corroborating the higher fertilizer-K apparent recovery efficiency in this treatment (Figure 2F). On the other hand, DSE-tomato interaction led to a significant accumulation of N, P, K, Ca, Mg, Fe (Figures 4A–F), and Zn (Figure 4H) compared with control plants when organic N source was supplied to tomato plants. Furthermore, inoculation with A105 resulted in higher Mn (Figure 4G) accumulation compared with control plants, but a similar Mn accumulation to the other inoculation treatments. N, P, and K contents increased by 24–33%, 33–39%, and 62–74%, respectively, corroborating the finely ground *C. ensiformis*-^15^N (Figure 2D), fertilizer-P (Figure 2G), and -K (Figure 2H) recovery efficiencies. Ca, Fe, Mn, and Zn contents increased by 25–41%, 72–178%, 17–31%, and 41–46%, respectively, while Mg content increased by 30%.

## Discussion

The DSE fungi tested in this study were obtained from wild rice (*O. glumaepatula*) collected from the Amazon region and identified by the ITS phylogeny to belong to order *Pleosporales* (suborder *Massarineae)* (A104 and A105) and an unknown taxon (A101) (Ribeiro, 2011; Vergara et al., 2017). DSE fungi were able to colonize the roots of tomato plants with no negative symptoms, even when the hyphae colonized the epidermis and the cortex of root plants and formed microsclerotia in the roots cells. Other studies have also reported colonization by DSE fungi in epidermis and cortex cells of tomato plants with no symptoms of pathologies (Andrade-Linares et al., 2011a; Mahmoud and Narisawa, 2013).

Similar to previous works on plant responses to inoculation with DSE fungi (Usuki and Narisawa, 2007; Diene et al., 2013; Mahmoud and Narisawa, 2013; Qin et al., 2017), the present study showed that inoculation with DSE fungi led to an increase in aboveground dry biomass of tomato plants supplied with an organic N source (finely ground *C. ensiformis*-^15^N). The same effect was not observed in plants treated with an inorganic N source (ammonium sulfate-^15^N). Conversely, Andrade-Linares et al. (2011b) reported that the DSE fungus *Leptodontidium orchidicola* was capable of increasing the shoot dry matter of tomato plants, as well as the fruit mass and glucose content, even in plants grown with an inorganic N source (NH_4_${\text{NO}}_{3}^{-}$).

A meta-analysis was performed from 18 studies carried out independently and confirmed that plants inoculated with carefully-selected DSE fungi responded positively when supplied with organic N sources, showing increases in dry matter and contents of N and P ranging from 26 to 106% (Newsham, 2011). Similarly, in the present study, which was carried out under greenhouse and non-sterile soil conditions, the DSE-tomato interaction was more efficient with use of N from an organic source. Increases were observed in the amount of nitrogen in the plant derived from finely ground *C. ensiformis*-^15^N, in the recovery efficiency of ^15^N, P, and K, in the contents of N, P, K, Ca, and Mg, plant height, leaf number, and total leaf area (A104), leading to increases in aboveground dry biomass ranging from 25 to 41% in comparison with control plants. Such results indicate that the DSE fungi A101, A104, and A105 can enhance tomato plant nutrition and growth when fertilized with an organic N source. Conversely, plants supplied with an inorganic N source only showed a positive inoculation effect for fertilizer-K recovery efficiency by plants, K content, and leaf area (A104), while other evaluated variables showed no effect. This suggested that the cultivar Santa Clara I-5300 does not need the fungus to absorb readily assimilable N as N-${\text{NH}}_{4}^{+}$, since breeding programs have frequently selected for plants more responsive to mineral fertilization (Borlaug and Dowswell, 1997). In a previous study performed under controlled hydroponic conditions, significant increases were not also observed in shoot N content using inorganic N sources (Torres-Júnior, 2014), although experimental conditions were different from those of the present study.

The ability of DSE fungi to promote growth and increase nutrients contents in tomato plants fertilized with finely ground *C. ensiformis-*^15^N suggests that these fungi are capable of degrading organic C, N, and P compounds, increasing macro and micronutrient availabilities to plants. Indeed, studies have reported that DSE fungi degrade organic compounds including cellulose, starch, proteins, lipids, amino acids, gelatin, urea, and pectin under *in vitro* conditions (Caldwell et al., 2000; Menkis et al., 2004; Mandyam et al., 2010; Surono and Narisawa, 2017). Additionally, DSE fungi promote the growth of several plant species supplied only with an organic N source (amino acids) or organic P source (phytate) under *in vitro* conditions (Usuki and Narisawa, 2007; Diene et al., 2013; Mahmoud and Narisawa, 2013; Surono and Narisawa, 2017). Recently, three isolates of *Phialocephala fortinii* led to the growth of *Asparagus officinalis* (L.) in agar medium supplied only with corn steep liquor (0.1%) or with corn steep liquor amended with inorganic nutrients (Surono and Narisawa, 2017). Although the preference in degrading a specific organic nutrient source can differ among species of DSE fungi (Diene et al., 2013; Surono and Narisawa, 2017), studies have suggested that DSE fungi are capable of mineralizing organic compounds containing N and P, making them available to plants. A recent study demonstrated that DSE fungi are capable of mineralizing organic P compounds under *in vitro* conditions (Della Monica et al., 2015). However, the mechanisms leading to an increase in the growth of plants inoculated with DSE fungi still need further research.

The highest K content and fertilized-K apparent recovery efficiency was observed for fertilization with ammonium sulfate-^15^N (for the fungus A104) and with finely ground *C. ensiformis*-^15^N (in all inoculation treatments), indicating that the DSE-tomato interaction was more responsive to K compare to other evaluated nutrients. This may be because K is the most required cation for plants and the most abundant cation in cytosol and plant dry matter (Marschner, 1995; Meurer, 2006). K acts in several physiological processes in plants, including photosynthesis, activation of more than 60 enzymatic systems, regulation of stomatal opening and closure, cell growth and elongation by generating cell turgor, and protein synthesis (Marschner, 1995).

During uptake K is transported from the soil solution to the root surface mainly by diffusion. Mass flow can significantly contribute to transport when the K concentration is high in the soil solution (Barber, 1995; Ruiz et al., 1999). However, diffusion is limited to very short distances from the root surface, usually around 1–4 mm (Meurer, 2006; Zeiger et al., 2017). Thus, the high K content and fertilizer-K apparent recovery efficiency observed under inoculation treatments strongly indicated that these fungi helped tomato plants absorb K through transposition of the K depletion zone and K interception in remote locations unattainable by the root surface.

Higher K and N contents are directly related to a greater total leaf area, since K acts on cell extension and N acts on leaf elongation, leading to a greater leaf area (Meiri et al., 1992; Chapman and Lemaire, 1993; Neto et al., 2007) and thus to greater plant exposition to sunlight (Neto et al., 2007). Contrary to previous studies (Melin, 1922; Wilcox and Wang, 1987; Stoyke and Currah, 1993; Mandyam et al., 2010; Diene et al., 2013), negative effects of inoculation with DSE fungi on plant growth were not observed.

Dark septate endophytic (DSE) fungi can also facilitate the uptake of micronutrients, including Fe, present in the soil (Bartholdy et al., 2001; Haselwandter, 2009). In this study, inoculation led to an increase in Fe content from 72 to 178%, and in Mn and Zn contents from 17–25% to 41–46%, respectively. These increases in Fe, Mn, and Zn contents under inoculation are related to a better use of these micronutrients in their source (soil, FTE BR12, and *C. ensifomis*-^15^N). Bartholdy et al. (2001) observed that DSE *Phialocephala fortinii* synthesized siderophore hydroxamate which led to an increase in Fe (III) uptake by host plants. When absorbed by plants, Fe acts on redox reactions in hemoproteins, such as cytochromes, and in non-heme proteins, such as ferredoxin (Dechen and Nachtigall, 2006). Ferredoxin and cytochromes are carriers of electrons during photosynthesis (Dechen and Nachtigall, 2006). Zn and Mn also act on enzymes involved in C metabolism. For instance, Zn is a structural component of carbonic anhydrase, which catalyzes CO_2_ dissolution (previous to its assimilation), and activates triphosphate dehydrogenase, an essential enzyme for glycolysis (Dechen and Nachtigall, 2006). Mn acts on photosystem II (in the Mn complex), which is responsible for water photolysis (Broadley et al., 2012). Currently, Mn has been recognized as an indicator of P acquisition efficiency by plants (Lambers et al., 2015). Thus, an increase in Fe, Mn, and Zn contents corroborates previous studies (Zhang et al., 2012, 2017; Ban et al., 2017) which suggested involvement of DSE fungi in photosynthetic activity by increasing chlorophyll levels, photochemical efficiency of photosystem II, net photosynthetic rate, stomatal conductance, and transpiration rate, leading to higher glucose (Andrade-Linares et al., 2011b) and soluble sugar contents (Vergara et al., 2017).

In general, tomato plants fertilized with finely ground *C. ensiformis*-^15^N showed significant differences among inoculation treatments, especially fungus A104, which had greater leaf number and area, and fungus A105, which had a higher Fe content. On the other hand, plants treated with ammonium sulfate-^15^N showed significant differences for K content and fertilizer-K apparent recovery efficiency, with an emphasis on fungus A104. These differences suggest that plant responses to inoculation with DSE depend on each fungus isolate, as it has already been observed in AMF inoculation (Mensah et al., 2015).

## Conclusion

Our findings indicated that tomato plants inoculated with DSE fungi acquired macro and micronutrients more efficiently, especially when an organic N source is used, resulting in increased plant growth. This finding was demonstrated using finely ground ^15^N-labeled *C. ensiformis* or ammonium sulfate-^15^N applied to soil. The A104 isolate seems to be the best option for inoculation of tomato cv. Santa Clara I-5300. However, a more detailed understanding of biochemical and molecular DSE-tomato interactions is still needed.

## Author contributions

CV, KA, SU, NS, FB, PM, LS, and JZ designed, performed experiments and analyzed data. CV, KA, FB, GX, and JZ conceived the experiments and wrote the paper. All authors read, edited, and approved the final manuscript.

### Conflict of interest statement

The authors declare that the research was conducted in the absence of any commercial or financial relationships that could be construed as a potential conflict of interest.
